# Epicardial and Pericoronary Adipose Tissue, Coronary Inflammation, and Acute Coronary Syndromes

**DOI:** 10.3390/jcm12237212

**Published:** 2023-11-21

**Authors:** Gianluigi Napoli, Valeria Pergola, Paolo Basile, Daniele De Feo, Fulvio Bertrandino, Andrea Baggiano, Saima Mushtaq, Laura Fusini, Fabio Fazzari, Nazario Carrabba, Mark G. Rabbat, Raffaella Motta, Marco Matteo Ciccone, Gianluca Pontone, Andrea Igoren Guaricci

**Affiliations:** 1 University Cardiologic Unit, Interdisciplinary Department of Medicine, Polyclinic University Hospital, 70124 Bari, Italy; gianluiginapoli@gmail.com (G.N.); pabas2304@gmail.com (P.B.); daniele.df93@gmail.com (D.D.F.); fbertrandino@gmail.com (F.B.); marcomatteo.ciccone@uniba.it (M.M.C.); 2 Department of Cardiac, Thoracic and Vascular Sciences and Public Health, University of Padua, 35122 Padua, Italy; valeria.pergola@yahoo.it; 3 Department of Perioperative Cardiology and Cardiovascular Imaging, Centro Cardiologico Monzino, IRCCS, 20138 Milan, Italy; andrea.baggiano@cardiologicomonzino.it (A.B.); saima.mushtaq@cardiologicomonzino.it (S.M.); laura.fusini@cardiologicomonzino.it (L.F.); fabio.fazzari@cardiologicomonzino.it (F.F.); gianluca.pontone@cardiologicomonzino.it (G.P.); 4 Department of Cardiothoracovascular Medicine, Azienda Ospedaliero Universitaria Careggi, 50134 Florence, Italy; n.carrabba@virgilio.it; 5 Division of Cardiology, Loyola University of Chicago, Chicago, IL 60611, USA; mrabbat@lumc.edu; 6 Edward Hines Jr. VA Hospital, Hines, IL 60141, USA; 7 Radiology Unit, University Hospital of Padova, 35128 Padua, Italy; raffaella.motta@unipd.it

**Keywords:** epicardial adipose tissue, pericoronary adipose tissue, coronary inflammation, acute coronary syndromes

## Abstract

Vascular inflammation is recognized as the primary trigger of acute coronary syndrome (ACS). However, current noninvasive methods are not capable of accurately detecting coronary inflammation. Epicardial adipose tissue (EAT) and pericoronary adipose tissue (PCAT), in addition to their role as an energy reserve system, have been found to contribute to the development and progression of coronary artery calcification, inflammation, and plaque vulnerability. They also participate in the vascular response during ischemia, sympathetic stimuli, and arrhythmia. As a result, the evaluation of EAT and PCAT using imaging techniques such as computed tomography (CT), cardiac magnetic resonance (CMR), and nuclear imaging has gained significant attention. PCAT-CT attenuation, which measures the average CT attenuation in Hounsfield units (HU) of the adipose tissue, reflects adipocyte differentiation/size and leukocyte infiltration. It is emerging as a marker of tissue inflammation and has shown prognostic value in coronary artery disease (CAD), being associated with plaque development, vulnerability, and rupture. In patients with acute myocardial infarction (AMI), an inflammatory pericoronary microenvironment promoted by dysfunctional EAT/PCAT has been demonstrated, and more recently, it has been associated with plaque rupture in non-ST-segment elevation myocardial infarction (NSTEMI). Endothelial dysfunction, known for its detrimental effects on coronary vessels and its association with plaque progression, is bidirectionally linked to PCAT. PCAT modulates the secretory profile of endothelial cells in response to inflammation and also plays a crucial role in regulating vascular tone in the coronary district. Consequently, dysregulated PCAT has been hypothesized to contribute to type 2 myocardial infarction with non-obstructive coronary arteries (MINOCA) and coronary vasculitis. Recently, quantitative measures of EAT derived from coronary CT angiography (CCTA) have been included in artificial intelligence (AI) models for cardiovascular risk stratification. These models have shown incremental utility in predicting major adverse cardiovascular events (MACEs) compared to plaque characteristics alone. Therefore, the analysis of PCAT and EAT, particularly through PCAT-CT attenuation, appears to be a safe, valuable, and sufficiently specific noninvasive method for accurately identifying coronary inflammation and subsequent high-risk plaque. These findings are supported by biopsy and in vivo evidence. Although speculative, these pieces of evidence open the door for a fascinating new strategy in cardiovascular risk stratification. The incorporation of PCAT and EAT analysis, mainly through PCAT-CT attenuation, could potentially lead to improved risk stratification and guide early targeted primary prevention and intensive secondary prevention in patients at higher risk of cardiac events.

## 1. Introduction

The burden of acute coronary syndrome (ACS) and its associated mortality have consistently increased over time [1,2]. Recent advancements in diagnostics and treatment have partially mitigated this upward trend. Coronary computed tomography angiography (CCTA) provides various tools with which to assess coronary artery disease, ranging from calcium scoring to coronary plaque analysis. These tools are utilized as comprehensive prognostic indicators (based on scores) [3,4,5,6,7,8,9,10,11,12,13,14,15,16,17]. 

Epicardial adipose tissue (EAT) and pericoronary adipose tissue (PCAT) contribute to cardiovascular risk in different ways. They have been shown to have a prognostic role in coronary artery disease (CAD) [18,19], to contribute to the development and progression of coronary artery calcification (CAC) and coronary plaque vulnerability [20,21,22], and to impact the inflammatory damage observed in coronary arteritis and myocardial infarction with non-obstructive coronary arteries (MINOCA) [23,24]. 

Emerging evidence suggests that the paracrine activity of EAT and PCAT is associated with coronary plaque instability [25,26]. It is clear that EAT and PCAT exhibit distinct biological properties which manifest differently under various circumstances. This review focuses on the emerging role of EAT in coronary inflammation, CAD development, plaque vulnerability, and ACS, with particular emphasis on its endocrine properties and the imaging modalities currently employed for its evaluation.

## 2. Imaging Evaluation of EAT and PCAT

EAT and PCAT, as well as thoracic fat, are commonly assessed using non-contrast computed tomography (CT) or cardiac magnetic resonance (CMR). Echocardiographic evaluation of EAT has also been described. Transthoracic echocardiography (TTE) is a readily available and user-friendly imaging modality for measuring EAT thickness. Typically, EAT is visualized in parasternal long-axis views as the hypoechoic space between the free wall of the right ventricle and the visceral layer of the pericardium during end-systole. Previous reports have indicated a median thickness of 7 mm [27,28]. However, the use of TTE for EAT evaluation is limited due to operator dependence, frequent poor acoustic windows, and poor correlation with CT-derived volume.

CMR can quantify EAT volume using black blood T1-weighted diastolic single-shot spin echo sequences [29] CMR allows for excellent visualization of the visceral and parietal pericardium, making EAT visualization straightforward, without the need for radiation exposure or contrast agents. However, the high cost, incompatibility with implanted devices, and claustrophobia in some patients are important limitations of this method.

**Figure 1:**
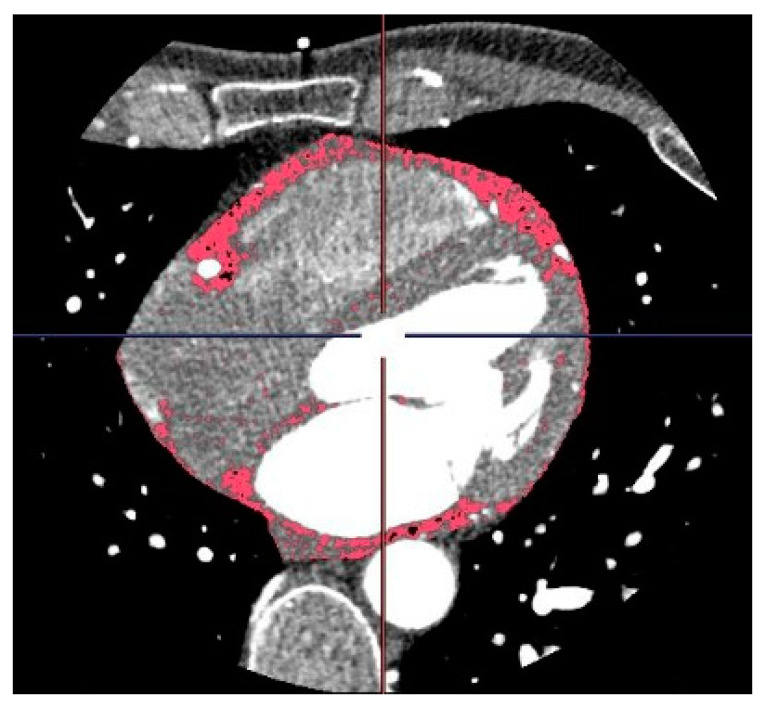
CT scan of a 49-year-old male patient depicting the epicardial adipose tissue (red) immediately internal to the pericardium.

**Figure 2:**
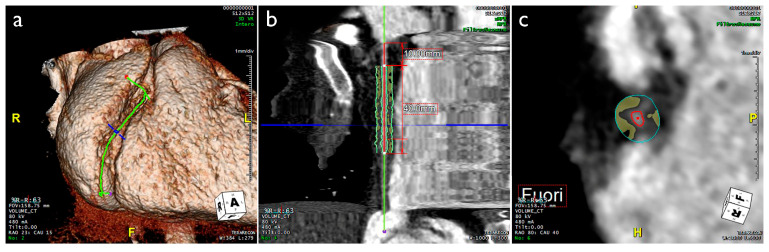
CT scan of a 39-year-old female patient. (**a**) 3D reconstruction of the heart, focusing on the middle segment of RCA. (**b**,**c**) PCAT distribution around RCA in longitudinal and ortogonal view, respectively (yellow spots). CT, computed tomography; PCAT, pericoronary adipose tissue; RCA, right coronary artery.

Currently, the epidemiological evidence on the role of EAT as a biomarker and predictor of cardiovascular disease (CVD) is mainly derived from CT imaging. In CT scan protocols, adipose tissue is identified by voxels with attenuation values between −30 and −190 Hounsfield Units (HU), depending on its normal or inflamed condition. The CT attenuation of PCAT represents the average attenuation, in HU, of the adipose tissue within the defined volume of interest. It reflects the balance between lipid and aqueous phases, with a well-established association between signal attenuation and adipocyte differentiation and size, primarily driven by intracellular lipid accumulation. Generally, a gradient from higher to lower attenuation has been observed from PCAT to EAT [18,30,31,32,33] (Figure 1 and Figure 2 and Table 1).

## 3. Pathophysiologic Role of EAT and PCAT

It is widely recognized that approximately 20% of the total ventricular mass is comprised of adipocytes [29,34,35,36]. EAT is situated in the atrioventricular and interventricular sulcus, surrounding the two appendages and the free walls of the atria. It maintains a consistent fat/muscle ratio even during hypertrophic changes [37,38,39]. EAT and PCAT, which originate from brown adipose tissue, share a close spatial and metabolic relationship with myocardial cells. Their role in immunological, inflammatory, metabolic, and vascular modulation of the heart has gained significant attention.

Under normal conditions, EAT exhibits various cardioprotective and metabolic properties. These include the release of free fatty acids (FFAs) as an energy source for the myocardium during periods of increased metabolic demand, the expression of the thermogenic protein UCP-1 in response to cold exposure, and the production of cardioprotective factors such as adrenomedullin. Adrenomedullin is a potent vasodilator and antioxidative peptide with anti-inflammatory and anti-atherogenic properties [40,41]. EAT also acts in a paracrine manner as a key regulator of vascular response during ischemia, sympathetic stimuli, and arrhythmia [42,43,44]. 

Firstly, EAT plays a role in initiating inflammatory signals in response to regional ischemia, leading to the release of interleukin (IL)-1β, IL-6, IL-6 soluble receptor, and tumor necrosis factor-α (TNFα), as well as a decrease in the secretion of adiponectin, an adipokine with anti-inflammatory and antiatherogenic properties [45,46]. Potential mechanisms involve the release of adipokines from PCAT, which may traverse the coronary wall through paracrine diffusion or direct release into the vasa vasorum, subsequently passing into the arterial wall in a vasocrine manner [40]. 

Secondly, increased EAT volume in obesity can disrupt ion channel properties, creating an arrhythmogenic substrate for atrial fibrillation (AF) [43]. Additionally, EAT has demonstrated heightened adrenergic activity in heart failure, as evidenced by higher concentrations of norepinephrine compared to plasma and the expression of catecholamine biosynthetic enzymes. This establishes a negative feedback loop, ultimately leading to functional and anatomical denervation of the heart [44].

## 4. Correlation between EAT, Coronary Inflammation, Coronary Flow Reserve, and Cardiovascular Risk

EAT consists of adipocytes, pre-adipocytes, ganglia, interconnecting nerves, immune cells, and inflammatory infiltrate, primarily macrophages [42,47]. The imbalance between pro-inflammatory M1 and anti-inflammatory M2 macrophages is observed in patients with CAD [48]. EAT, which is in close proximity to myocardiocytes, can release pro-inflammatory and pro-atherogenic substances directly into the coronary lumen under pathological conditions. The transcriptome of EAT encodes inflammatory cytokines, affecting its pro-atherogenic characteristics, especially in unfavorable metabolic conditions like diabetes [49,50,51,52,53,54]. Radiographic fat density correlates with adipocyte enlargement, while CT attenuation is inversely related to adipocyte size. Higher CT attenuation in EAT indicates the presence of enlarged adipocytes and infiltration of pro-inflammatory M1 macrophages, indicating inflammation [48]. CT attenuation of the surrounding PCAT has prognostic significance in CAD, as elevated attenuation is associated with reduced coronary flow reserve [18,19,32], while a pericoronary fat attenuation index (pFAI) exceeding a specific threshold is predictive of both all-cause and cardiac mortality [55]. Utilizing the pFAI could potentially improve risk assessment and guide targeted prevention strategies for individuals at higher risk [56].

**Figure 3:**
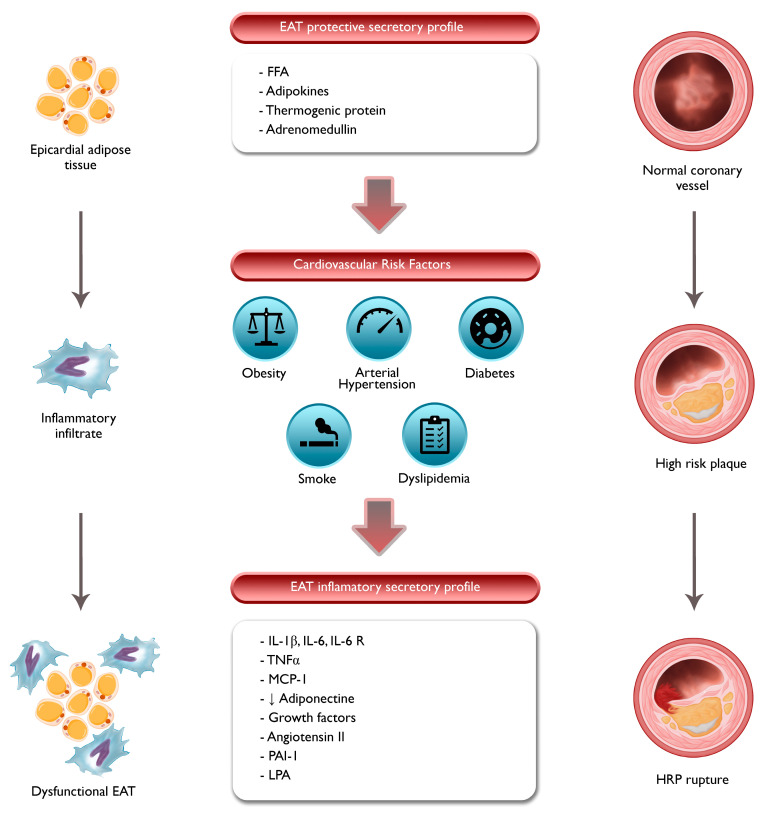
Interaction between EAT, PCAT, and atherogenesis. EAT, epicardial adipose tissue; FFA, free fatty acid; HRP, high-risk plaque; IL, interleukin; LPA, lysophosphatidic acid; MCP, monocyte chemoattractant protein; PAI, plasminogen activator inhibitor; TNF, tumor necrosis factor. EAT, located around the coronary arteries, secretes various bioactive molecules such as adipokines, cytokines, and FFAs. In the presence of cardiovascular risk factors, EAT undergoes inflammation, which is characterized by increased infiltration of immune cells. Dysfunction of PCAT and its effects on the coronary arteries can contribute to the initiation and progression of atherosclerosis, leading to the development of HRP.

PCAT has shown a positive association with the presence, amount, and progression of CAC, independent of total body fat [20,21,22] Even after considering conventional cardiovascular risk factors and inflammatory markers, the volume of EAT remains a significant predictor of CAC burden [21]. This may be attributed to the development of an inflammatory micro-environment, lower levels of anti-atherogenic cytokines, and the secretion of inflammatory substances such as ILs and monocyte chemoattractant protein-1 (MCP-1). Consequently, dysfunctional EAT/PCAT promotes inflammatory infiltration, leading to tissue changes that are reflected in the attenuation observed through CT imaging (Figure 3).

Moreover, EAT volume has previously been associated with an increased CVD risk profile in a directly proportional manner [57,58,59]. However, in advanced heart failure (HF), reductions in EAT mass have been observed due to the lipolytic activity induced by natriuretic peptides. In end-stage HF, lower EAT volume predicts a poorer prognosis [60]. This suggests a U-shaped association between EAT volume and CVD risk across the entire spectrum of ejection fraction. Furthermore, a recent meta-analysis has confirmed the prognostic role of EAT/PCAT volume, demonstrating positive associations with obstructive CAD and major adverse cardiovascular events (MACE) [57].

## 5. EAT/PCAT Activity Overcomes Systemic Inflammatory Markers in ACS

Various biomarkers, including microRNA (miRNA), IL-6, B-type natriuretic peptide (BNP), cardiac troponin I (c-TnI), adiponectin, adipocyte fatty acid-binding protein (A-FABP), high-sensitivity C-reactive protein (hs-CRP), and lipocalin-2, have been associated with coronary inflammation and ACS. However, their lack of specificity limits their clinical utility [61]. Standard non-invasive approaches fail to accurately identify coronary inflammation, endothelial dysfunction, and high-risk coronary plaques (HRP) [62,63].

C-TnI is released from myocardiocytes following cardiac injury, not only in the case of myocardial infarction but also in other pathological conditions such as HF, myocarditis, sepsis, pulmonary embolism, and renal failure [64,65]. BNP, primarily a marker of ventricular stretch, increases in response to ventricular hypertrophy and myocardial infarction, playing a role in volume homeostasis and ventricular remodeling in HF [66,67,68,69]. Adiponectin, an anti-inflammatory and anti-atherogenic adipokine, has been inversely associated with endothelial dysfunction and obstructive CAD, but its plasma level is influenced differently by factors such as body mass, insulin resistance, and triglycerides [67]. A-FABP, expressed in adipocytes and macrophages, is involved in glucose and lipid metabolism and has been linked to the severity of coronary atherosclerosis, although its expression is similarly influenced by insulin resistance, hypertension, and HF [70,71]. Lipocalin-2, an inflammatory marker found in various tissues, including adipose tissue, has been associated with atherosclerosis development and CAD severity, and its levels increase in response to factors such as body mass, type 2 diabetes mellitus (T2DM), and insulin resistance [67,72,73]. Elevated levels of circulating hs-CRP and IL-6 in patients with stable angina have been correlated with HRP, MACE, and hospitalization for HF [74,75,76], with higher CRP levels observed in patients with acute myocardial infarction (AMI) compared to those with stable CAD [77,78]. However, while these biomarkers can detect both systemic and coronary inflammation, they lack specificity and do not provide information regarding the localization of coronary plaques. 

On the other hand, CT imaging of PCAT inflammation can specifically identify HRP, with progressively higher attenuation observed from stable CAD to ACS [79,80]. PCAT CT attenuation does not correlate with standard circulating inflammatory biomarkers, as shown in a post hoc analysis of the SCOT-HEART study. This suggests that CT attenuation can identify coronary inflammatory status and active plaques that cannot be traced by systemic markers [81]. Furthermore, studies indicate that mean PCAT attenuation is higher in patients with myocardial infarction compared to those with stable CAD, suggesting phenotypic changes in the entire coronary tree during ACS, which is consistent with findings from intracoronary imaging [79,80,82]. 

## 6. Correlation between PCAT, Plaque Vulnerability, and ACS

EAT plays a role in plaque instability and ACS through its paracrine regulation of coronary vessels. HRP are prone to rupture and exhibit positive remodeling without significant lumen narrowing. Standard imaging methods may not accurately assess non-obstructive, but high-risk, plaques [83]. However, innovative approaches using 18F-sodium fluoride (18F-NaF) positron emission tomography (PET) uptake and PCAT attenuation on CT have shown promise [84,85,86]. 

PCAT may contribute to atherogenesis, plaque instability, and ACS (Figure 3) [25,26,87]. Studies have demonstrated that increased PCAT CT attenuation is associated with atherosclerotic coronary segments and plaque rupture in non-ST elevation myocardial infarction (NSTEMI). Moreover, EAT attenuation has been shown to be directly proportional to the probability of future AMI [88]. PCAT CT attenuation is higher in culprit lesions compared to non-culprit lesions and stable CAD controls. Indeed, it has emerged as an independent predictor of culprit lesions in patients with multivessel CAD. 

The exact mechanisms underlying the relationship between PCAT and HRP development are not fully understood. It remains unclear whether inflammatory signals from coronary vessels with HRP influence PCAT or whether PCAT exerts vasocrine regulation through inflammatory pathways. PCAT, which is closer to the coronary arteries than EAT, has a greater number of small pre-adipocytes, with a higher adipogenic gene expression profile than mature adipocytes as result of the paracrine actions of the inflammatory environment implicated in CAD [31]. Whereas EAT catches both paracrine and autocrine signals, PCAT is primarily impacted by paracrine signaling from the blood vessels. Proposed mechanisms include the release of growth factors by adipocytes, secretion of angiotensin II and plasminogen activator inhibitor-1 by EAT, and the release of lysophosphatidic acid (LPA) promoting smooth muscle cell proliferation (Figure 3) [89,90]. 

Further research is needed in order to fully comprehend the complex interactions between PCAT, coronary inflammation, and plaque vulnerability. These findings highlight the potential of PCAT attenuation for CT and 18F-NaF PET uptake as non-invasive markers of coronary inflammation and plaque instability [86].

Increased myocardial oxidative stress leads to the release of oxidation messenger products, which can affect the secretory profile of EAT and its role in heart disease [91]. EAT, in response to oxidative stress, releases adiponectin, an adipokine with antioxidant properties, to protect the myocardiocytes and endothelium from oxidative stress. Thus, the phenotype of PCAT could be the result, rather than the cause, of underlying heart disease [91].

Regardless of the underlying mechanism, these findings suggest that PCAT can be used as a non-invasive tool to detect HRP, enhancing the identification of high-risk patients who could benefit from aggressive primary prevention strategies.

### Autoptic Evaluation of EAT and Risk of Sudden Cardiac Death

PCAT attenuation on CT is emerging as a highly sensitive method to evaluate coronary inflammation, with biopsy-proven results supporting its validity [91]. In an autoptic study of 139 cross-sections of the left anterior descending (LAD) artery obtained from 16 patients, the ratio between plaque volume and media thickness was directly proportional to EAT volume and macrophage infiltration of PCAT. PCAT volume was also proportionate to the extension of a lipid core and to the inflammatory infiltration of atherosclerotic plaque [92].

In a post mortem comparison of CT data vs. autoptic findings in 116 human hearts, patients with significant CAD showed higher extension of EAT and PCAT. Moreover, both the thickness and volume of EAT significantly correlated with the grade of epicardial coronary artery obstruction, even after adjusting for confounding factors [93].

Sudden cardiac death (SCD) describes a natural death, usually occurring within an hour of the beginning of symptoms after rapid loss of consciousness if appropriate resuscitation techniques are not performed promptly [94]. It has a multifaceted etiology in which a preexisting cardiovascular disease, usually undetected, culminates in cardiac rhythm abnormalities and cardiac arrest. Since visceral adipose tissue as a CVR factor in SCD has sparked interest, EAT has been investigated as marker of coronary atherosclerosis. In a forensic, retrospective case–control study, EAT was an independent predictor of SCD [95]. Moreover, in a retrospective analysis of 321 autopsy cases, Hogea et al. demonstrated that patients with silent myocardial infarction (SMI) had higher prevalence of EAT at the left circumflex artery and the LAD artery, which were found to be independent predictors of SMI upon multivariate analysis [96].

## 7. Correlation between PCAT and Coronary Arteritis

Perivascular adipose tissue (PVAT) is located at the outermost boundary of the arterial wall and has been suggested to play a role in the pathogenesis of vasculitis. Studies have hypothesized that PVAT may play a triggering role in the development of vasculitis, and isolated peri-adventitial inflammation has been associated with an increased likelihood of developing giant cell arteritis. This suggests a primary role of PVAT in the pathogenesis of systemic vasculitis [23]. 

In Takayasu arteritis (TAK), a type of inflammatory arteritis affecting the aorta and its branches, the density of periaortic adipose tissue (PAAT) and PCAT has been quantified. Patients with TAK showed higher PAAT and PCAT density compared to patients with CAD or controls, independently of other factors. PAAT and PCAT density were closely related to markers of disease activity in TAK, indicating a potential role of PAAT and PCAT in the pathogenesis of TAK. PCAT density was also identified as an independent predictor of coronary inflammation, as visualized by 68Ga-DOT PET imaging [24]. 

Kawasaki disease (KD), a type of acute inflammatory vasculitis primarily affecting medium-sized elastic arteries, particularly the coronary arteries, is a major cause of acquired heart disease in children. Coronary artery aneurysms and inflammatory cell infiltration have been observed in KD, and adipokines have been suggested to play a central role in its pathogenesis. A meta-analysis showed that resistin and adiponectin levels were significantly elevated in KD patients with coronary artery lesions compared to those without coronary involvement [97,98,99].

While further research is needed in order to fully understand the relationship between PCAT and arteritis, it is reasonable to assume that the inflammatory microenvironment surrounding coronary vessels, including the contribution of PCAT through paracrine secretion, plays a causative role in the development and progression of coronary damage. Future studies are needed to investigate the role of PCAT in regulating vessel inflammation and to explore potential therapeutic approaches in this field.

## 8. Correlation between PCAT and MINOCA

MINOCA is diagnosed when invasive coronary angiography reveals a patent coronary tree in a patient with AMI [100]. The prevalence of MINOCA is 1–13% of all patients with a clinical diagnosis of AMI, and several potential etiologies exist. After initial negative angiography, MINOCA may be reclassified as type 1 or type 2 AMI due to mechanisms of myocardial ischemia; the first represents a plaque-induced event where plaque rupture or erosion with superimposed thrombus develops spontaneous recanalization. On the contrary, type 2 AMI arises from non-plaque-induced conditions due to reduced blood supply and oxygen imbalance, such as spontaneous coronary artery dissection (SCAD), embolism, vasospasm, and microvascular disease [101]. Mechanisms causally connecting plaque rupture/erosion with PCAT, as well as endothelial dysfunction and EAT, have been described previously in this document. Furthermore, emerging evidence currently exists regarding the contributory role of EAT in other type-2 MINOCA [102].

### 8.1. Spontaneous Coronary Artery Dissection

SCAD is defined as a non-traumatic, non-atherosclerotic separation of the layers of the arterial wall, resulting in the creation of a false lumen. The role of inflammatory infiltrate in the pathogenesis of SCAD is still debated, and a causative or healing role has not been conclusively determined. Some reports suggest a causative role of eosinophilic infiltrates in coronary wall injury, leading to the expansion of intramural hemorrhage through the release of cytotoxic products and stimulation of aberrant neovascularization [103,104]. On the other hand, a large pathology case series has suggested that periadventitial inflammation in SCAD is time-dependent and proportional to the time elapsed between symptom onset and death, indicating a healing response of the inflammatory infiltrate in SCAD [105]. 

Regardless of its contribution, an inflammatory environment is consistently present in SCAD, involving both the vessel wall and the PCAT bidirectionally [106]. In vivo detection of vascular inflammation would be useful, and several studies have evaluated the role of PCAT CT attenuation in this context. One study failed to show a significant difference in median PCAT attenuation between SCAD patients and controls, potentially due to the healing process and resolution of ACS with invasive or medical treatment [107]. However, another study demonstrated that elevated PCAT density on CT performed within 48 h of coronary angiography had a higher prevalence than the imaging of dissection identified by angiography [108]. Additionally, a cohort study showed a significant association between PCAT CT attenuation, wall motion abnormality, and initial elevated troponin levels in patients with high-degree stenosis due to SCAD or atherosclerosis [106]. 

While invasive coronary angiography is the first-line examination for patients presenting with ACS and suspected SCAD, CT may play a role, especially in those with early presentation and normal initial troponin levels. PCAT CT attenuation can provide valuable information, particularly when small dissections may not be detectable due to the spatial resolution limitations of CT [109].

### 8.2. Vasospastic Angina 

The endothelium modulates vascular tone through the release of relaxing factors (mainly prostaglandins and nitric oxide) and hyperpolarizing factors. The former mediates the relaxation of large arteries (i.e., epicardial coronary arteries), while the latter plays a key role in regulating the resistances of small arteries by opening calcium-activated potassium channels and subsequently hyperpolarizing the membranes of vascular smooth muscle cells (VSMCs) [110]. Multiple studies have attempted to identify the mechanism behind coronary vasospasm, and different pathological reports have described extensive adventitial inflammation near the spastic coronary artery [111,112]. Such inflammatory environment seems to mediate VSMCs’ hypercontraction by the influx of Ca^2+^ through L-type Ca-channel as an initial trigger, which is functionally up-regulated via PKC and a GTPase-Rho-dependent mechanism [110]. Moreover, Ohyama et al. recently demonstrated increased PCAT volume at the site of higher lumen reduction in spastic coronary segments, which suggests the involvement of PCAT in the inflammatory context leading to the spasm [113].

As previously described, 18F-FDG PET/CT is able to detect perivascular inflammation, with signal intensity directly proportional to macrophage density and microcalcifications [114]. As expected, ECG-gated 18F-FDG PET/CT recently showed significantly increased FDG uptake in patients with vasospastic angina, which significantly decreased during the follow-up period [115]. Numerous studies have suggested that PVAT regulates the vascular bed in a paracrine way, with opposite effects in the peripheral and coronary districts due to different proteomic responses secondary to local and systemic factors. Owen et al. previously demonstrated that biological factors are released from PCAT potentiate coronary vasoconstriction, with a directly proportional expression of RhoA (2.9-fold) and calpastatin (1.6-fold) to total fat volume, leading to increased function of VSMCs’ Ca^2+^ channels, H_2_O_2_-sensitive K^+^ channels, or mediators regulating these channels [116]. Notably, inhibition of Ca^2+^ channels with nifedipine or diltiazem is able to neutralize this effect.

These data suggest a key role of PCAT in regulating vascular tone in the coronary district beyond endothelial function and systemic mediators, highlighting the potential detrimental effects of dysregulated PCAT in type 2 MINOCA.

## 9. Future Perspectives: Application of Artificial Intelligence

The term artificial intelligence (AI) refers to the application of computing methods to tasks that ordinarily call for human intellect. AI subfields, such as machine learning (ML), are increasingly used in cardiovascular imaging; ML models can now be employed to merge clinical data with AI-derived information for personalized risk stratification [117]. CCTA-derived quantitative measures have previously been included in ML models, which have outperformed qualitative or quantitative high-risk plaque characteristics alone in terms of outcome prediction [118,119].

Deep learning (DL), a subset of ML, generates assumptions directly from input data through multilayered artificial neural networks. Recently, Driessen et al. applied DL to CT-derived fractional flow reserve (CT-FFR) software (HeartFlow FFR_CT_ version 2.7, Redwood City, CA, USA) to aid in identifying the coronary lumen border and generating analyses of hybrid computational fluid dynamics, demonstrating the greater diagnostic performance of CT-FFR compared to CCTA for stenosis evaluation and PET for ischemia detection [120]. Moreover, the ML method demonstrated advantageous application in PET/TC as well, allowing for automated CAC scoring from low-dose CT-attenuation images routinely acquired during PET protocol, with outstanding results compared to the reference standard of manual quantification [117]. More recently, Commanduer at al. compared DL to manual analysis of EAT, finding a high level of accuracy with the automated analysis, which also performed better than human evaluation in terms of identifying non-calcified plaque. Additionally, DL took about 1.57 s per patient, compared to 15 min for professional readers [121]. A boost ensemble ML method has recently been evaluated to compare EAT volume and CFR in non-contrast CT and PET. The ML composite risk score significantly enhanced the risk reclassification of impaired myocardial flow reserve compared to EAT volume or CAC score alone [122]. Thus, AI-integrated approaches are now not only feasible, but have been proven to enhance CVR assessment without additional radiation exposure, greatly reducing the time needed for manual measurements.

Interestingly, radiomics, a new frontier of imaging evaluation through data-characterization algorithms, has recently been applied in PCAT analysis with interesting results [123]. Oikonomou et al. used PCAT radiomic features to train a random forest ML model to create a patient-level “fat radiomic profile.” This indicator demonstrated incremental utility for MACE prediction beyond traditional CCTA-based risk categorization when assessed in 1575 SCOT-HEART study participants [124]. Therefore, the texture and geometry-based parameters of PCAT may outperform PCAT CT attenuation in terms of discriminating high-risk plaque, even though the lack of standardized methodology in the radiomic workflow, complex manual delineation, and the need for highly experienced staff are important limitations to its widespread adoption [125]. However, the combination of radiomics and PCAT CT attenuation could further enhance the identification of patients at risk of acute coronary events.

## 10. Conclusions

Vascular inflammation is the main driver of ACS, but standard noninvasive approaches such as exercise treadmill testing (ETT) and perfusion imaging have failed to precisely identify coronary inflammation and subsequent endothelial dysfunction. Existing noninvasive methods, like 18F-NaF-PET, which is well standardized in terms of identifying coronary inflammation, require complex imaging protocols and processing. Additionally, circulating biomarkers that are widely used lack adequate specificity. As a result, the noninvasive detection of HRP and the dysfunctional endothelium remains challenging.

PCAT undeniably plays a key role in coronary inflammation and the development of HRP due to the bidirectional interconnection between epicardial fat and the coronary vessels. PCAT evaluation through CT attenuation has emerged as a promising method for predicting MACE, with demonstrated relationships between PCAT volume/density and coronary flow reserve (CFR), CAC, and cardiovascular risk. Furthermore, PCAT attenuation has recently shown the ability to predict plaque rupture in AMI and accurately identify culprit lesions in patients with multivessel CAD. Given its proximity to the vascular wall, PCAT has also been hypothesized to play a triggering role in coronary vasculitis and MINOCA.

AI has been progressively applied in cardiovascular risk stratification, and ML models incorporating CCTA-derived quantitative measures have demonstrated incremental utility in predicting MACE compared to plaque characteristics alone. Therefore, the evaluation of PCAT appears to be a safe, valuable, and sufficiently specific noninvasive method for accurately identifying coronary inflammation and subsequent HRP, with its results having been proven both via biopsy and in vivo.

While speculative, these pieces of evidence pave the way for a fascinating future strategy for cardiovascular risk stratification. It is conceivable that the assessment of EAT status may be incorporated into risk stratification tools, enabling early targeted primary prevention and intensive secondary prevention in patients at higher risk of cardiac and all-cause mortality.

## Figures and Tables

**Table 1:** Imaging modalities by which to assess adipose tissue.

	TTE	CCTA	CMR
Availability	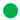 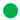 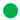	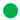 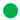 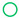	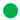 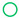 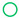
Cost	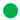 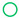 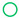	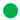 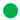 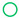	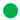 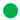 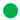
Lack of iodine contrast use	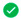	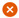	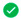
Lack of ionizing radiation exposure	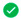	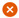	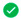
Reproducibility	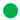 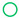 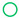	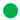 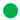 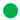	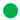 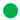 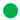
Spatial resolution	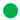 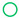 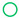	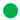 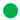 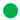	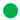 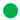 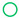
3D volume data	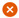	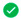	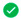
AT thickness	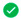	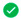	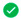
AT area	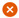	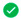	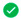
AT volume	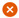	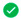	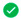
AT attenuation	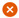	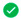	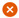
AT radiomic profile	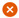	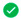	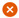

AT, adipose tissue; CCTA, coronary computed tomography angiography; CMR, cardiac magnetic resonance; TTE, transthoracic echocardiography. 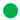

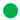

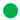
, high; 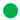

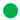

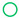
, medium; 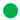

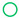

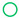
, low; 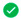, condition satisfied/measure allowed; 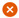, N/A.

## Data Availability

Not applicable.

## Abbreviations

A-FAB	adipocyte fatty-acid-binding protein
ACS	acute coronary syndrome
AF	atrial fibrillation
AI	artificial intelligence
AMI	acute myocardial infarction
BNP	B-type natriuretic peptide
C-TnI	cardiac troponin I
CAC	coronary artery calcification
CAD	coronary artery disease
CCTA	coronary CT angiography
CFR	coronary flow reserve
CMR	cardiac magnetic resonance
CVD	cardiovascular disease
CT	computed tomography
CT-FFR	CT-derived fractional flow reserve
DL	deep learning
EAT	epicardial adipose tissue
ETT	exercise treadmill testing
FFAs	free fatty acids
HF	heart failure
HRP	high-risk coronary plaques
hs-CRP	high-sensitivity C-reactive protein
HU	Hounsfield units
IL	interleukin
KD	Kawasaki disease
LAD	left anterior descending
LPA	lysophosphatidic acid
MACE	major adverse cardiovascular events
ML	machine learning
MCP-1	monocyte chemoattractant protein-1
MINOCA	myocardial infarction with non-obstructive coronary arteries
MiRNA	microRNA
NSTEMI	non-ST-segment elevation myocardial infarction
PAAT	periaortic adipose tissue
PCAT	pericoronary adipose tissue
PET	positron emission tomography
pFAI	pericoronary fat attenuation index
PVAT	perivascular adipose tissue
SCAD	spontaneous coronary artery dissection
SCD	sudden cardiac death
SMI	silent myocardial infarction
T2DM	type 2 diabetes mellitus
TAK	Takayasu arteritis
TNFα	tumor necrosis factor-α
TTE	transthoracic echocardiography
VSMCs	vascular smooth muscle cells
18F-NaF	18F-sodium fluoride
